# Silver nanoparticles enhance the sensitivity of temozolomide on human glioma cells

**DOI:** 10.18632/oncotarget.13503

**Published:** 2016-11-22

**Authors:** Ping Liang, Hongming Shi, Weiguo Zhu, Qunfeng Gui, Ya Xu, Jianfeng Meng, Xiaoyuan Guo, Zhuang Gong, Huaqun Chen

**Affiliations:** ^1^ Department of Neurology, Yancheng Hospital Affiliated to Southeast University, Yancheng, 224001, China; ^2^ Department of Neurology, Zhongda Hospital Affiliated to Southeast University, Nanjing, 210009, China; ^3^ Department of Nephrology, The First People's Hospital of Yancheng Affiliated to Nantong University, Yancheng, 224001, China

**Keywords:** silver nanoparticles (AgNPs), glioma cells, temozolomide (TMZ), chemotherapy

## Abstract

Glioblastoma multiforme (GBM) continues to be associated with a dismal prognosis despite aggressive treatment. Significant efforts are being made to develop new nanotechnology-based therapeutic and diagnostic agents. Nanoparticles can act directly on cancer cells or as drug carriers to enhance the cancer therapeutic effect. In this study, we investigated the effect of silver nanoparticles (AgNPs) on human glioma U251 cells and its role in the combinational use with Temozolomide (TMZ), an imidazotetrazine derivative of the alkylating agent dacarbazine, against glioma cells. AgNPs were synthesized in the sodium citrate system and the mean size were 26 nm in diameter. The AgNP particles showed dose-dependent cytotoxicity on U251 cells. They also showed the ability to enhance the drug-sensitivity of TMZ on U251 cells. Our results revealed that AgNPs could have a potential application in enhancing chemotherapy for glioma.

## INTRODUCTION

Glioblastoma multiforme (GBM), one of the most common malignant tumors in the central nervous system (CNS), remains a lethal disease with poor prognosis. The treatment of GBM is multimodality in which surgical resection, radiotherapy and chemotherapy play important roles [1, 2]. Among them, chemotherapy remains the gold standard for patients who can not tolerate surgical procedure or relapse after surgery. However, most glioma cancer eventually develops multidrug resistance (MDR) to current chemotherapeutic drugs that limits the effectiveness of treatment results [3, 4].

To circumvent the cancer drug resistance, strategies have been focus on developing novel therapeutic agents and enhancing cancer drug delivery efficacy. Direct delivery of drug to the tumor site had been proposed and extensively studied. In recent years, scientists were able to synthesize nanoscale, biodegradable and biocompatible drug delivery systems with the advanced nanotechnology. Such accomplishment has made it possible to deliver anticancer drugs using nanoparticles as delivery vehicles [5] .

There are many types of nanocarriers that have been developed to deliver a variety of drugs. The most common nanocarriers are polymeric nanoparticles, solid lipid nanoparticles and liposomes [6–8]. These nanocarriers have shown excellent characteristics as drug delivery system. They reduced non-specific cellular uptake, prolonged the lifetime of drug in systemic circulation. They can control drug release, improve drug targeting ability. Nanocarriers can also be generated as, multidrug encapsulation for treatments that require multi-regimen.

In recent years, a number of chemotherapeutic drugs have been successfully encapsulated in nanoparticles for cancer treatment. The average size of these nanoparticles ranges from 50 to 150 nm. Studies have demonstrated the strength of using nanoparticle delivery system to tackle cancer drug resistance [5]. There are several clinical trials are in progress using nanoparticle-encapsulated anti- cancer drugs. Some of the nanoparticle encapsulated anti-cancer drugs are currently available on the market.

Silver nanoparticles (AgNPs) have showed the property of anti-cancers. There were studies show that AgNPs can enter cells by endocytosis [9]. Inside the cells, AgNPs localize in the perinuclear region and endo-lysosomal membrane compartment [10]. Cytotoxicity assay indicated that AgNPs has cytotoxicity whose potency is similar to that of Ag ions [11]. Some researches showed that AgNPs can enter the mitochondria and affect cells’ respiration, generating reactive oxygen species (ROS), therefore induced cell apoptosis [12–14]. Besides, AgNPs could cause DNA damage and induce p53 protein expression [15]. Some studies also showed that the function of vascular endothelial growth factor (VEGF) could be affected by AgNPs [16]. These findings confirmed that AgNPs had anti-tumor properties that could be used as an alternative for cancer treatment and for treating angiogenesis-related diseases [17].

Temozolomide (TMZ) is a second-generation imidazotetrazine derivative, which exerts its cytotoxic effects by methylation of specific DNA sites [19]. Although it is effective in the treatment of glioblastoma, drug resistance limits its successful application. It was known that AgNPs have differential sensitivity to normal cells (e.g. human lung fibroblast cells), as compared to cancer cells (e.g. human glioblastoma cells) [18]. In the present study, we investigate the cytotoxicity of AgPNs on glioma cells and explore whether AgNPs can enhance the sensitivity of TMZ against glioma. We demonstrated that the combination of AgNPs and TMZ has potent anti-glioma effect and could be explored to developing into novel chemotherapeutic agents for cancer treatment.

## RESULTS

### Characteristics of AgNPs

**Figure 1:**
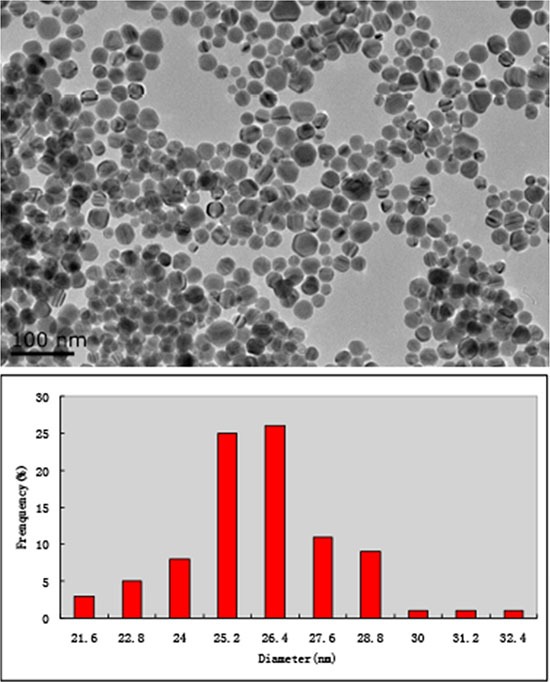
TEM micrograph of AgNPs AgNPs are heterogeneous spheres (**A**) with the mean sizes of 26 nm in diameters (**B**).

AgNPs were prepared from the sodium citrate system by using AgNO_3_ and NaBH_4_. Under transmission electron microscopy (TEM), AgNPs was found to be heterogeneous spheres with a diameter ranging from 21–32 nm. They were uniformly distributed. The mean diameter of AgNPs was 26 nm (Figure 1).

### Cytotoxicity of AgNPs

Our previous studies showed that AgNPs have a definite dose-dependent toxicity on cells. We usually regard 1/5 of IC50 as a suitable concentration, because of better biocompatibility and little toxic effect on normal cells. In the preliminary experiments, U251 cells were incubated with different concentrations of AgNPs. We found necrotic and apoptotic cells when AgNPs concentration was greater than 92 μmol/L. Most of the cells grow normally when the AgNPs concentration was less than 46 μmol/L, the 1/5 of IC50 of AgNPs. Therefore 46 μmol/L of AgNPs was used in the following experiments.

### Uptake of AgNPs by U251 cells

**Figure 2:**
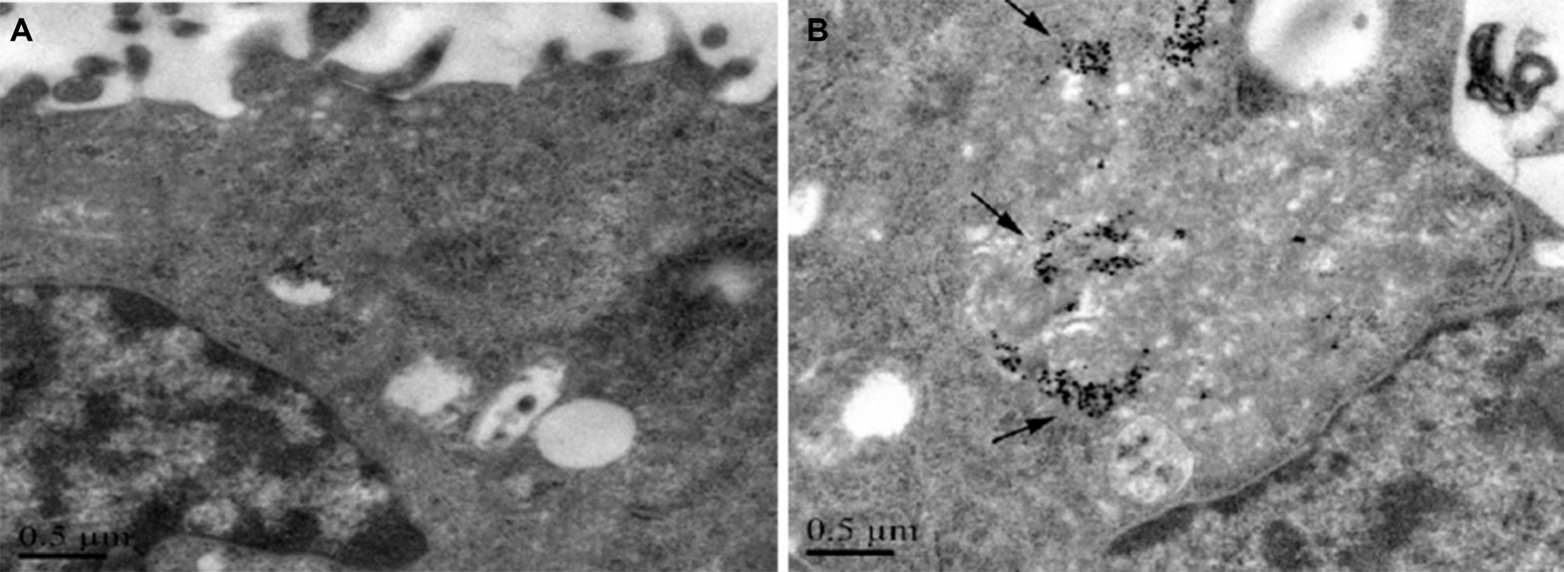
TEM micrograph of U251 cells treated with AgNPs AgNPs were found distributed inside the cells. (**A**) untreated, (**B**) AgNPs treated.

TEM micrographs show that AgNPs distributed inside the cells. AgNPs containing endosomes were mainly seen near the cell membrane. This result indicated that AgNPs might enter the cells through membrane endocytosis. In addition to the nanoparticles-containing endosomes, autophagic cellular vacuoles filled with structures resembling mitochondria and electron dense contents of unknown origin were also observed inside the AgNPs treated cells. A few nanoparticles were seen in the nucleus of the treated cells. (Figure 2).

### Colony formation assay

**Figure 3:**
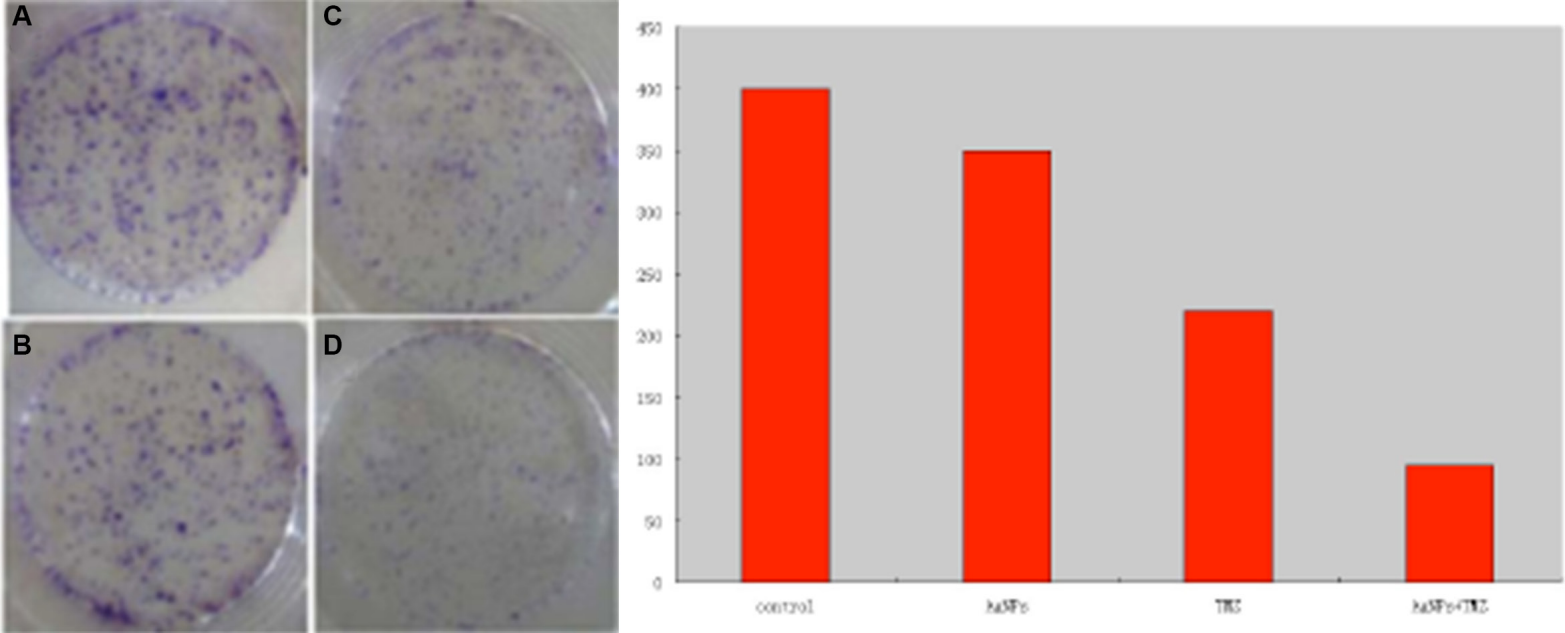
Images of colony-forming assays (**A**) control; (**B**) cells incubated with AgNPs; (**C**) cells incubated with TMZ; (**D**) cells incubated with AgNPs + TMZ; (**E**) Quantitative results of A, B, C, and D.

U251 cells form colony upon culture in Petri dishes. Under the treatment with AgNPs at the concentration of 46 μmol/L, the cells’ colony forming ability decreased to around 80% compared to cells without AgNPs treatment. Similarly, TMZ treatment repressed U251 cells’ colony forming ability. Treatment of U251 cells with the combination of TMZ and AgNPs, the number of survival colonies significantly decreased (Figure 3). This result indicated that AgNPs could enhance the effect of TMZ-induced cell death.

### AgNPs induced cell apoptosis and significantly enhanced TMZ sensitivity in inducing cell apoptosis

**Figure 4:**
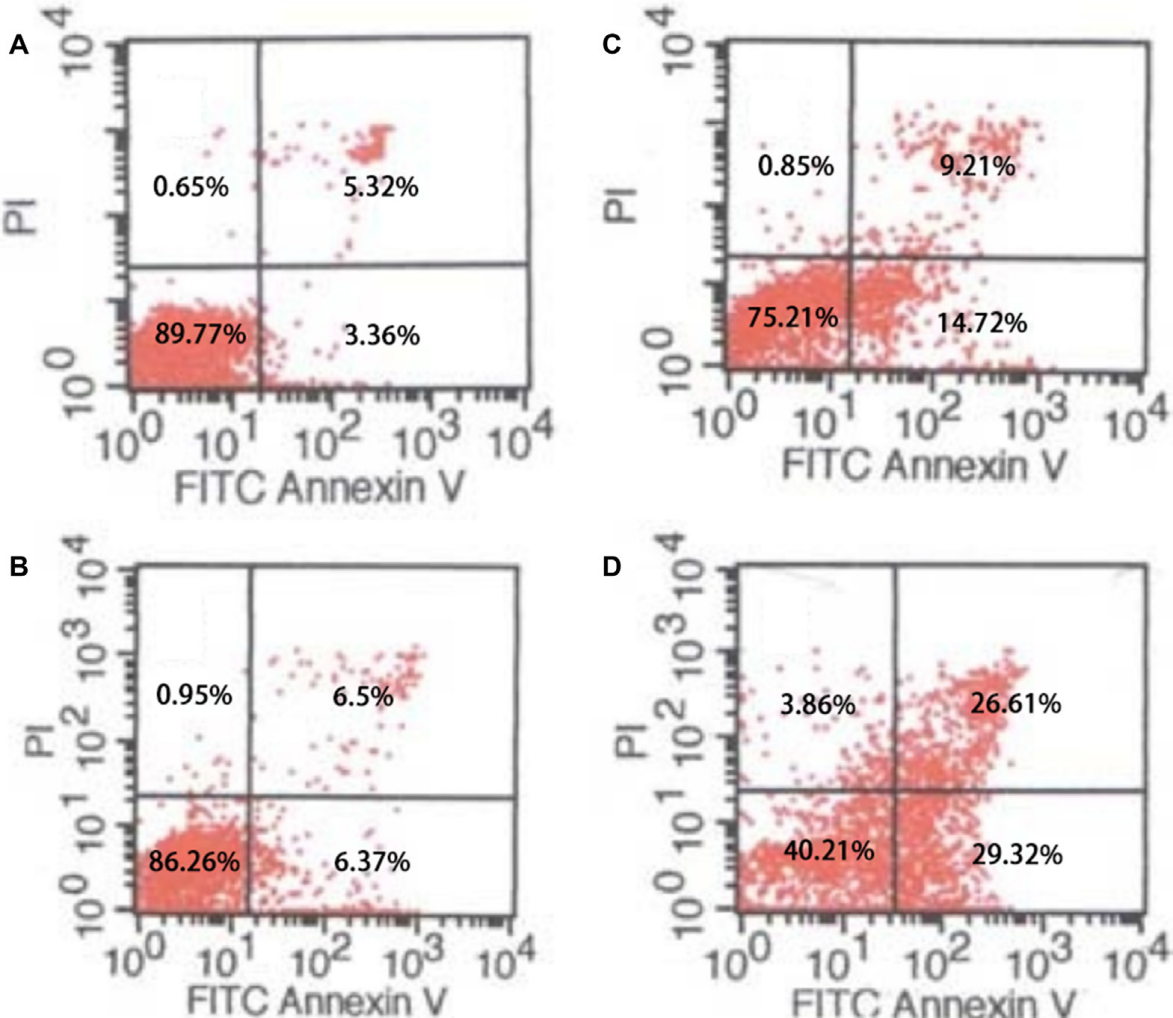
Flow cytometry Cell apoptosis of U251 cells treated with AgNPs and TMZ. (**A**) control; (**B**) cells incubated with AgNPs; (**C**) cells incubated with TMZ; (**D**) cells incubated with AgNPs + TMZ.

The above results showed that TMZ combined with AgNPs significantly decrease the cell survival rate. To understand whether the decreased cell survival was associated with apoptosis, PI and Annexin-V-FITC staining was performed and analyzed using flow cytometry. U251 cells were treated with AgNPs, TMZ or combination of both. The results showed although AgNPs or TMZ alone can increase cell apoptosis to certain degree. The apoptosis rate increased much more in cells treated by the combination of AgNPs and TMZ (*P* < 0.05) (Figure 4). These results demonstrated that combination of AgNPs and TMZ further induce apoptosis in cancer cells.

### AgNPs induced cell cycle arrest and significantly enhanced TMZ sensitivity in inducing cell cycle arrest

**Figure 5:**
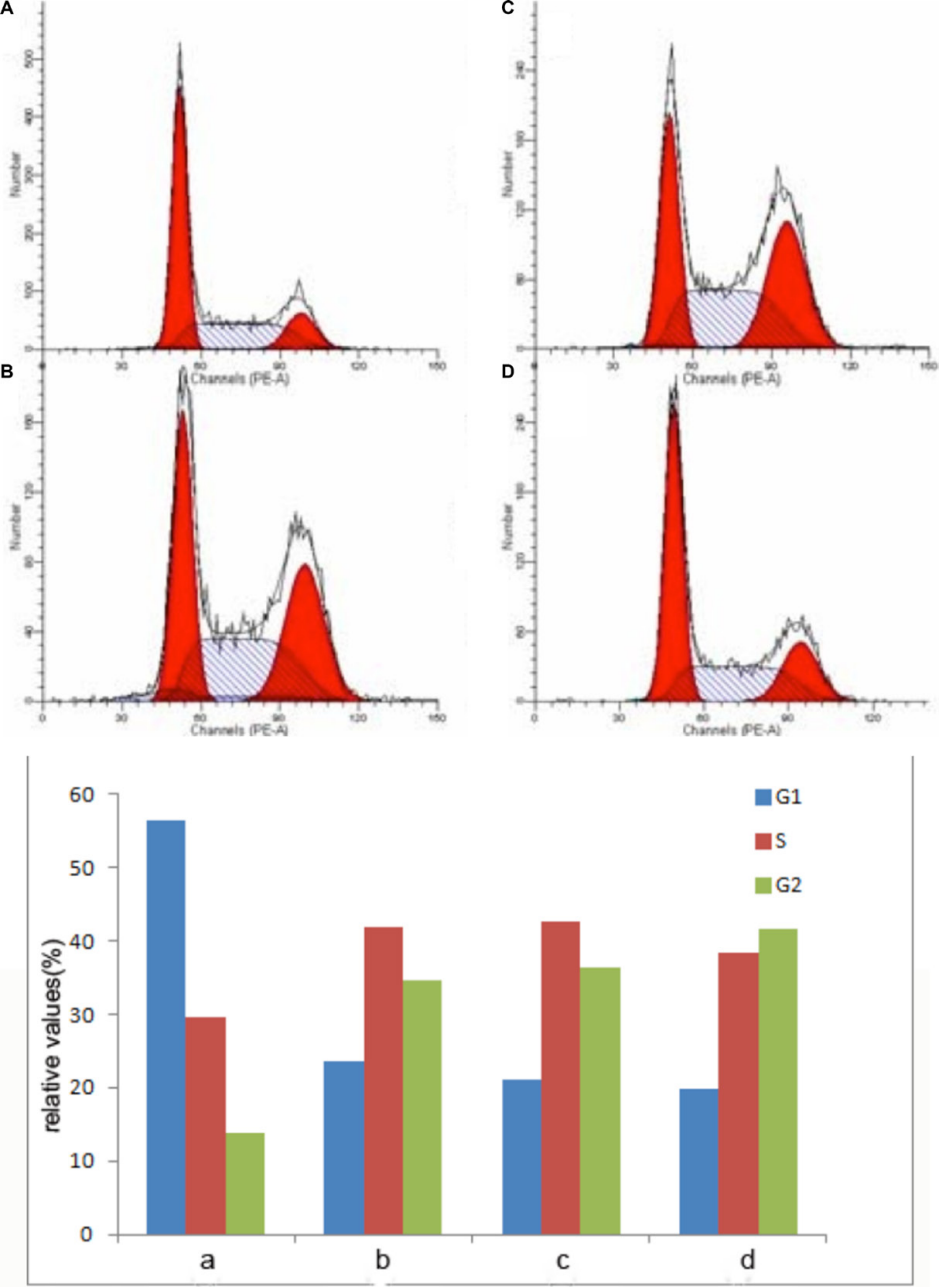
Effect of AgNPs on cell cycle distribution before and after TMZ in U251 cells (**A**) control; (**B**) cells incubated with AgNPs; (**C**) cells incubated with TMZ; (**D**) cells incubated with AgNPs + TMZ.

Analysis of the cell cycle showed that both AgNPs and TMZ alone could induce G2/M arrest. The combinational use of AgNPs with TMZ further induce G2/M arrest (Figure 5). This result indicated that AgNPs can induce cell cycle arrest and enhance the sensitivity of TMA in inducing cell cycle arrest. This result might also explain why more cells could enter apoptosis phase after incubated with AgNPs.

## DISCUSSION

It was know that AgNPs can induce dose-dependent cytotoxicities that include DNA damage and oxidative stress that can result in cell death. Gliomas are sensitive to agents that cause DNA damage and oxidative stress. AgNPs have differential sensitivity to normal cells (e.g. human lung fibroblast cells), as compared to cancer cells (e.g. human glioblastoma cells) [18]. Studies have shown the cytotoxic effect of AgNPs on a variety of cancer cells, e.g. glioblastoma cells and breast cancer cells [21–25]. AgNPs has been used as a radiosensitizer in the treatment of glioma [25].

In this study, experiments were carried out to study the cytotoxic effect of AgNPs on human glioma cells. Factors that affect the cytotoxicity of AgNPs include the shape and size of particles, the surface charge and capping agent. We found that AgNPs with the size of diameter around 26 nm can be uptake into cells and released as silver ions, which interfered with the intracellular repair process. Our results indicated that AgNPs have high cytotoxicity against glioma cells at a very low concentration (46 μmol/L) that have little effect on normal cells. AgNPs induced DNA damage and apoptosis. The cytotoxic effect of AgNPs was concentration-dependent and was enforced when combined use with chemotherapeutic agent TMZ.

As one of the most common and lethal primary malignant tumors in the central nervous system, the prognosis of GBM is very poor. Current regimes for GBM include surgical resection and aggressive treatment with radiotherapy and chemotherapy. Although progress has been made, the results are still not satisfying. In recent years, significant efforts are devoted in the development of nanotechnology-based therapeutic agents. The present study provides a rational for using AgNPs to develop into therapeutic agents for GBM. In summary, our studies show that AgNPs have selective cytotoxicity against cancer cells, particularly on glioma cells at doses that are nontoxic to normal cells. It has a dose-enhancement effect in the combinational use with TMZ. These results implicated the potential use of AgNPs as a therapeutic agent for GMB therapy.

## MATERIALS AND METHODS

### AgNPs synthesis

AgNPs was synthesized according to published protocol. Briefly, 0.1 M AgNO3 (0.5 mL) was added into 40 mL deionized water, and then mixed with freshly prepared 0.02 M NaBH4 (1 mL) aqueous solution with vigorous stirring. A solution of 1% sodium citrate (10 mL) was added during the reduction. The solution was allowed to stir for an additional 30 sec [20].

The prepared AgNPs was centrifuged and the supernatant was discarded. AgNPs were then dispersed into Fetal bovine serum (FBS, Sigma Corp. Ltd, Shenzhen, China ) and transferred into Dulbecco's modified eagle's medium (DMEM, Sigma Corp. Ltd) (FBS: DMEM = 1:9). The samples received 20 Gy dose of X-rays irritation for sterilization. The prepared AgNPs were examined by transmission electron microscope (TEM, JEM-2010, JEOL Ltd., Tokyo, Japan).

### Cell culture

The human glioma U251 cells were supplied by Shanghai Institute of Cell Biology and cells were maintained in presence of Glutamine and nonessential amino acids (NEAA), supplemented with 10% FBS (Sigma Corp. Ltd). Cell cultures were incubated at 37°C and equilibrated in 5% CO2 and air.

### MTT assay

U251 cells were transferred into 96-well micro-plates at a density of 1 × 10^3^ cell/well. At the end of 24 h culture, cells were cultured with different concentrations of TMZ or AgNPs for another 24 h. After that, the medium was removed. Then 20 μl of 3-(4,5-Dimethylthiazol-2-yl)-2,5-diphenylterazolium bromide (MTT, Sigma) (5 mg/L) was added to each well, and placed at 37°C for 4 h. Dimethyl sulfoxide (DMSO, 150 μl ) was added to each well to dissolve the dark blue crystal products. Absorbance was measured at a wavelength of 570 nm using a multi-well spectrophotometer (Bio-Rad, Hercules, USA).

### Colony formation assay

The cells were seeded in 1 × 10^4^ per well and cultured in 60 mm Petri dishes at 37°C with 5% CO2 in a humidified incubator (three replicates). Twenty-four hours later, AgNPs were added into the medium and the concentration of AgNPs was kept in (1/5 of IC50) μmol/L. Cells were incubated with AgNPs for another 24 h. Then cells were incubated with TMZ with a concentration of 31 μmol/L based on previous MTT assays for another 24 h. After that, cells were washed twice with PBS and re-cultured in the medium containing 10% FBS for 14 d. Colonies were fixed with methanol, treated with Giemsa stain (Sigma Corp. Ltd), and counted using a microscopy.

### Transmission electron microscopy of cells treated with AgNPs

Cells were treated with AgNPs 46 μmol/L for 24 h, washed well to remove unbound AgNPs. Cells were fixed in 2.5% gluteraldehyde for 2 h and washed in phosphate buffer. Post fixation was done in 1% osmium tetroxide for 1 h. Cells were washed further in phosphate buffer and dehydrated in a series of alcohol for 15 min each (50%, 70%, 80%, 95% and 100%). Cells were further treated with propylene oxide (30 min), propylene oxide-resin mixture (overnight) and pure resin (48 h). Embedding was done in BEEM capsules using pure Spurrs low viscosity resin at 80°C for 48 h. Ultrathin sections were taken using Reichert Jung Ultratome and negatively stained. The stained sections were observed under a transmission electron microscopy (TEM, JEM-2010, JEOL Ltd, Tokyo, Japan)

### Apoptosis analysis

An *in situ* Apoptosis Detection Kit was used for Annexin V-FITC binding and PI staining (Sigma Corp. Ltd). Following incubation with AgNPs or AgNPs + TMZ for 24 h, 2 × 10^5^ cells from each group were harvested and washed with PBS and resuspended in binding buffer (10 mM HEPES, 140 mM sodium chloride, 1.8 mM calcium chloride, pH 7.4). Then fluorescein-conjugated Annexin V-FITC (1 μg/ml) and PI reagent (5 μg/ml) were added into cell suspensions. Then the cells were incubated in the dark for 15 min at room temperature and analyzed immediately by flow cytometry (BD Sciences, San Diego, USA).

### Cell cycle analysis

After incubated with AgNPs for 24 h, cells were incubated with TMZ for 24 h. Then these cells were harvested after treatment and were fixed in 70% ethanol at 4°C for 24 h. Before analysis, cells were washed once in PBS, digested with 500 U/mL RNase for at least 30 min at 37°C and then stained with PBS containing 50 μg/ml PI for 30 min. Analyses were performed with a flow cytometer (BD Sciences). Cell Cycle distributions were calculated on DNA plots by BD FACS Diva software (Verity Software House).

### Statistical analysis

Sigma plot was used for statistical analysis. Each experiment was performed for at least 3 times. Statistical differences between control and experimental groups were calculated using Student's *t* test. *P* < 0.05 was considered statistically significant.
